# Analysis of Clinical Related Factors of Neonatal Hand-Foot-Mouth Disease Complicated With Encephalitis

**DOI:** 10.3389/fneur.2020.543013

**Published:** 2020-11-12

**Authors:** Yanling Fang, Chaowei Lian, Dali Huang, Liping Xu

**Affiliations:** ^1^Zhangzhou Hospital Affiliated to Fujian Medical University, Zhangzhou, China; ^2^Zhangzhou Zhengxing Hospital, Zhangzhou, China

**Keywords:** neonates HFMD, prognosis, risk factors, clinical symptoms, CSF examination, inflammatory factors

## Abstract

**Objective:** To explore the clinical related factors of neonatal hand-foot-mouth disease (HFMD) complicated with encephalitis.

**Method:** The neonatal HFMD complicated with encephalitis treated in our hospital from July 2015 to July 2020 was taken as the object of study. According to the NBNA score at discharge, the patients were divided into normal group and abnormal group. The clinical symptoms, auxiliary examination and prognosis of the two groups were compared.

**Result:** (1) General condition: there was no significant difference in sex, age, duration of fever, treatment time and etiological test between the two groups (*P* > 0.05). (2) Clinical symptoms and signs: there was significant difference in abnormal consciousness between the two groups (*P* < 0.05). However, there was no significant difference in skin rash, respiratory system symptoms, digestive system symptoms, signs of high intracranial pressure, increased muscle tone and weakening of primitive reflex (*P* > 0.05). (3) Auxiliary examination: the number of white blood cells and the level of cytokines (CK-BB, UCH-L1) in cerebrospinal fluid (CSF) in the group with abnormal NBNA score were significantly higher than those in the group with normal NBNA score (*P* < 0.05). The serum IgM level in the abnormal NBNA score group was higher than that in the normal NBNA score group, and the serum IgG level in the abnormal NBNA score group was lower than that in the normal NBNA score group, and the difference was statistically significant (*P* < 0.05). The abnormal rate of Craniocerebral MRI in abnormal NBNA score group was higher than that in normal NBNA score group, and there was significant difference between the two groups (*P* < 0.05). There was no significant difference in the levels of protein, sugar, chloride, lactate dehydrogenase, and MMP-9 in CSF and the abnormal rate of amplitude integrated EEG (aEEG) between the two groups (*P* > 0.05). (4) The prognoses of patients with normal and abnormal NBNA score are good, and there are not significantly differences in the prognosis between the two groups (*P* > 0.05).

**Conclusion:** (1) Neonatal HFMD complicated with encephalitis occurs more than 10 days after birth, there is no obvious abnormality in male and female, the vast majority of newborns have febrile symptoms, rash is not its specific manifestation, and most of them are atypical. (2) The positive rate of HFMD-related virus detected in CSF of neonatal HFMD is high. For newborns with abnormal consciousness, CSF examination should be accomplished in time, which has certain clinical significance for early diagnosis and treatment of severe newborns. (3) The increase of white blood cell count and cytokines (CK-BB, UCH-L1) in CSF of neonatal HFMD complicated with encephalitis has a certain clinical reference value for early diagnosis and identification of severe newborns. (4) There is a certain humoral immune disorder in newborns with HFMD complicated with encephalitis, but the overall prognosis is better due to the protective effect of maternal IgG.

## Introduction

HFMD is an acute infectious disease caused by enteroviruses. Coxsackie A group 16 (CA16) and enterovirus 71 (EV71) are the most common infections. These viruses can cause aseptic meningitis, brainstem encephalitis and neurogenic pulmonary edema as the main causes of death (1). The peak period of the disease is from May to July every year, which is more common in preschool children, especially in the age group under 3 years old (2). Because of the special age of newborns, there is little contact with the outside world, HFMD is easy to be ignored, and there are few reports about neonatal HFMD at home and abroad. However, among the patients admitted to our hospital in recent years, neonatal HFMD is increasing year by year (3), but there are differences in clinical manifestations and laboratory indicators between preschool children and newborns, which may be because newborns are protected by maternal antibodies.

To assess inflammatory damage in the brain of newborns with HFMD, we analyzed three important proteins involved in neuroinflammation. Brain creatine kinase isoenzyme (CK-BB), which exists in brain neurons and astrocytes, is a sensitive marker of neuronal injury and has high specificity for judging brain neuronal injury. There is also a close correlation between the elevated degree of CK-BB in various nervous system diseases and the degree of brain injury and prognosis. Under normal circumstances, the content of CK-BB in human blood and CSF is very low. When brain tissue lesions lead to brain cell edema and damage, CK-BB will be released into the cerebrospinal fluid, resulting in an increase in the concentration of CK-BB in the cerebrospinal fluid (4).

Matrix metalloproteinase 9 (MMP-9), also known as gelatinase B, has a molecular weight of about 92KD and can be secreted by neutrophils, monocytes, vascular endothelial cells, smooth muscle cells and other cells (5). In infectious diseases of the central nervous system, MMP-9 participates in a variety of pathophysiological activities, which can cooperatively degrade a variety of extracellular matrix components, promote inflammation and destroy the blood-brain barrier (6).

Ubiquitin carboxyl-terminal hydrolase L1 (UCH-L1) is a neuronal cytoplasmic protein. Its expression level in human brain is about 50 times higher than that in other tissues, especially in large neurons such as Purkinje cells, brainstem nucleus neurons and basal ganglion neurons, accounting for 1~2% of the total human brain protein (7). Because of its high expression and specificity, UCH-L1 can be used as a marker of neuronal injury. At present, it has been found that its expression is increased in a variety of nervous system diseases, and the level of serum UCH-L1 in children with hypoxic encephalopathy is closely related to the prognosis (8, 9).

The purpose of this study was to explore the clinical features of neonatal HFMD complicated with encephalitis through the comparative analysis of clinical manifestations, CSF routine and inflammatory factor levels (CK-BB, MMP9, UCH-L1), serum immunoglobulin levels (IgM, IgG, IgA), craniocerebral MRI, aEEG, and prognosis of neonatal HFMD complicated with encephalitis.

## Materials and Methods

### Selected Objects and Grouping

A total of 46 neonates with HFMD complicated with encephalitis admitted to our hospital from July 2015 to July 2020 were selected, including 26 males and 20 females. These cases were confirmed by routine CSF and CSF HFMD-related virus examinations (including 41 cases of HFMD universal nucleic acid and 5 cases of Coxsackie virus), which met the diagnostic criteria for HFMD complicated with encephalitis (10).

The NBNA scale is routinely used for neurological assessment when the newborns are discharged from the hospital. The total score is 40 points, the score ≥35 is considered normal, and the score <35 is considered abnormal. The experimental groups were divided into normal score group and abnormal score group. There were 29 cases with normal NBNA score and 17 cases with abnormal NBNA score. There was no significant difference in age, sex, course of disease, and other general data between the two groups (*P* > 0.05), which was comparable, as shown in Table 1. All the family members of the newborns who participated in the study were aware of the process, purpose and significance of the study, and agreed to participate, which was approved by the Medical Ethics Committee of our hospital.

**Table 1:** Comparison of clinical symptoms and signs of neonatal HFMD with encephalitis.

**Factors**	**NBNA≥35 Group (*n* = 29)**	**NBNA<35 Group (*n* = 17)**	**X2/*t***	***P***
Sex male (male/female)	17/12	9/8	0.141	0.708
Age **(**d)	15.21 ± 4.41	14.71 ± 4.13	0.380	0.706
Fever duration(d)	3.82 ± 1.75	4.12 ± 1.45	0.575	0.568
Treatment time**(**d)	14.41 ± 1.01	15.06 ± 1.25	1.907	0.063
Rash	11	6	0.032	0.858
Breathing (shortness of breath, apnea)	9	6	0.088	0.766
Digestion (anorexia, vomiting, abdominal distension)	11	9	0.983	0.322
Jaundice	6	3	0.063	0.802
Disturbance of consciousness (drowsiness, irritability, screaming, poor reaction)	8	10	4.391	0.036^*^
High intracranial pressure (front fontanelle tension, fullness, eminence)	10	8	0.712	0.399
Increased muscular tension	9	5	0.013	0.908
Original reflection attenuation	5	4	0.269	0.604

* *P < 0.05, which is statistically significant*.

All the family members of the newborns who participated in the study were aware of the process, purpose and significance of the study, and agreed to participate, which was approved by the Medical Ethics Committee of our hospital (Ethics approval No.: Zhangyilun 20180211).

### Exclusion Criteria

(1) newborns who have recently used drugs that can enhance or inhibit human immunity; (2) patients with immune diseases such as tuberculosis; (3) other nervous system diseases, genetic and metabolic diseases, congenital brain dysplasia, craniocerebral trauma, congenital cerebrovascular dysplasia and other diseases that affect the development of nervous system.

### Methods

#### General Data

The sex, age, clinical symptoms, and signs of the children were recorded, including the duration of fever, rash, reaction, respiratory symptoms, digestive system symptoms, state of consciousness, signs of high intracranial pressure, muscle tone, original reflection and so on.

#### Laboratory Examination

CSF (4 ml) was collected before starting treatment in all newborns, in which 2 ml CSF was examined by routine biochemical examination, and 2 ml CSF was centrifuged and the supernatant was stored in refrigerator at−80°C for testing. The concentrations of CK-BB, MMP-9, and UCH-L1 in CSF were detected by double antibody sandwich ELISA method. (the kit is provided by Sigma Company of the United States catalog. The catalog number of the CK-BB, MMP-9 and UCL-L1 were C9983, RAB0372, and RAB1766, respectively). 2 ml of venous blood was routinely extracted on admission for all newborns, which was centrifuged and stored at−20°C for later use. Immunoglobulin (IgM, IgG, IgA) in the blood was detected by means of serum immunoturbidimetry.

#### Neuroimaging Examination

Craniocerebral MRI was selected to evaluate the prognosis of the children, and the axial, sagittal, and coronal scans were performed by Signa EXCITE HD3.0T superconducting magnetic resonance imaging made by GE Company of the United States.

#### aEEG Examination

Within one week of onset, aEEG was performed, and aEEG was monitored and recorded by American NicoletOne Monitor brain function monitor. According to the international 10: 20 standard electrode placement system, 9-lead system was selected, which were forehead (Fp1,Fp2), temporal region (T3, T4), central area (C3, C4), occipital area (O1,O2), top of head (CZ), grounding electrode GND (midpoint of Fp1 and Fp2), reference electrode REF (the midpoint of CZ and PZ). The impedance setting is <15 k Ω and continuous monitoring is carried out for at least 8 and 10 h.

#### Neonatal Behavioral Neurological Assessment

The NBNA rating scale was used for evaluation (Chinese Neonatal Behavioral nerve score of 20 items). Professional nurses were selected to score newborns before discharge, including behavioral ability, active muscle tension, primitive reflex, passive muscle tension, and general assessment, which were divided into 20 items, each item was scored 2 points, a total of 40 points, the score less than 35 points may be abnormal, and more than 35 points is normal. The score was positively correlated with the neurological function of the newborns.

### Statistical Analysis

SPSS 20.0 Statistical Software was used for data analysis. The measurement data was normally distributed and represented by mean and standard deviation(SD). After homogeneity of variance analysis, *t* test was used for comparison between the two groups. Counting data were expressed as rates, chi-square test was used, and *P* < 0.05 was statistically significant.

## Results

### General Situation

Forty six cases of neonatal HFMD complicated with encephalitis were evaluated by NBNA before discharge. There were 29 cases with normal NBNA score, including 17 males (58.6%) and 12 females (41.4%). There were 17 patients with abnormal NBNA score, including 9 males (52.9%) and 8 females (47.1%). There was no significant difference in gender between the two groups. The age of onset of the normal NBNA score group was (15.21 ± 4.41) days, and the treatment time was (14.41 ± 1.01) days. The age of onset of the abnormal NBNA score group was (14.71 ± 4.13)d, and the treatment time was (15.06 ± 1.25)d. There was no significant difference in the age of onset and treatment time between the two groups (*P* > 0.05), as shown in the Table 1.

### Etiological Analysis

Among the 46 cases, the positive rate of CA16 was 5 cases (10.9%), including 2 cases with normal NBNA score and 3 cases with abnormal NBNA score. The positive rate of general nucleic acid of HFMD virus was 41 cases (89.1%), including 27 cases with normal NBNA score and 14 cases with abnormal NBNA score. The positive rates of different kinds of viruses in normal and abnormal NBNA groups were tested by chi-square test. The results showed that there was no significant difference between normal and abnormal groups (X^2^ = 1.279, *P* > 0.05), as shown in the Table 2.

**Table 2:** Etiological analysis of neonatal HFMD complicated with encephalitis.

**Group**	**CA16**	**HFMD universal nucleic acid**
NBNA ≥35 Group	2	27
(*n* = 29)		
NBNA<35 Group	3	14
(*n* = 17)		
X^2^	1.279	
*P*	0.258	

### Clinical Symptoms and Signs

Among the 46 cases, all cases had fever symptoms, the fever duration in the normal NBNA score group was (3.82 ± 1.75)d, the fever duration in the abnormal NBNA score group was (4.12 ± 1.45)d, and there was no significant difference between the two groups. Among the 46 cases, rash was found in 17 cases (36.9%), respiratory symptoms (shortness of breath, apnea, periodic breathing) in 15 cases (32.6%), digestive symptoms (anorexia, vomiting, abdominal distension) in 20 cases (43.5%), jaundice in 9 cases (19.6%), intracranial hypertension (front fontanelle tension, fullness, eminence) in 18 cases (39.1%), and muscular tension in 14 cases (30.4%). The original reflex decreased in 9 cases (19.6%). There was no significant difference in the proportion of the above clinical symptoms between the normal group and the abnormal group in NBNA score. Abnormal consciousness (drowsiness, irritability, screaming, poor reaction) was found in 18 cases (39.1%), including normal NBNA score group (*n* = 8) and abnormal NBNA score group (*n* = 10). There was significant difference between the two groups (*P* < 0.05), as shown in the Table 1.

### Routine Examination of CSF

The leucocyte count of CSF in the abnormal NBNA score group (139.35 ± 72.25 10∧6 /L), was significantly higher than that in the normal NBNA score group(96.17 ± 48.66 10∧6 /L), and the difference was statistically significant (*P* < 0.05). There was no significant difference in the levels of protein, sugar, chloride, and lactate dehydrogenase in CSF between the abnormal NBNA score group and the normal group (*P* > 0.05), as shown in the Table 3.

**Table 3:** Routine comparison of CSF in newborns with HFMD complicated with encephalitis.

**CSF**	**NBNA≥35 Group**	**NBNA<35 Group**	***t***	***P***
	**(*n* = 29)**	**(*n* = 17)**		
WBC(10∧6 /L)	96.17 ± 48.66	139.35 ± 72.25	2.367	0.022^*^
Protein(g/L)	0.55 ± 0.20	0.72 ± 0.34	1.834	0.080
Sugar(mmol/L)	2.19 ± 0.42	2.29 ± 0.24	0.913	0.366
Chlorides(mmol/L)	126.21 ± 5.71	125.00 ± 7.13	0.631	0.556
Lactate dehydrogenase(U/L)	34.41 ± 8.56	33.27 ± 5.78	0.474	0.638

* *P < 0.05, which is statistically significant*.

### Cytokine Level of CSF

The level of CK-BB in CSF was (9.02 ± 2.75) U/L in the abnormal NBNA score group, which was significantly higher than that in the normal NBNA score group (7.10 ± 2.53) U/L. The level of UCH-L1 in CSF was (1.06 ± 0.53) ng/ml in the abnormal NBNA score group, which was significantly higher than that in the normal NBNA score group (0.78 ± 0.32) ng/ml, with statistically significant difference between groups (*P* < 0.05). There was no significant difference in the level of MMP-9 in CSF between the two groups (*P* > 0.05), as shown in the Table 4.

**Table 4:** Comparison of cytokine levels in CSF of newborns with HFMD complicated with encephalitis.

**CSF**	**NBNA≥35 Group**	**NBNA<35 Group**	***t***	***P***
	**(*n* = 29)**	**(*n* = 17)**		
CK-BB (U/L)	7.10 ± 2.53	9.02 ± 2.75	2.411	0.020^*^
MMP9 (ug/L)	39.03 ± 10.45	44.35 ± 12.28	1.563	0.128
UCH-L1(ng/ml)	0.78 ± 0.32	1.06 ± 0.53	2.215	0.032^*^

* *P < 0.05, which is statistically significant*.

### Immunoglobulin Content

The serum IgM level in the abnormal NBNA score group was(0.57 ± 0.20) g/L, which was significantly higher than that in the normal NBNA score group (0.45 ± 0.15)g/L. The serum IgG level in the abnormal NBNA score group was (6.55 ± 1.52) g/L, which was significantly lower than that in the normal NBNA score group (7.61 ± 1.41) g/L. The difference between groups was statistically significant *(P* < 0.05). The IgA levels of the two groups of newborns were lower, and the IgA levels of most of the newborns were below 0.26 g/L as shown in the Table 5.

**Table 5:** Comparison of serum immunoglobulin levels in newborns with HFMD and encephalitis.

**Serum**	**NBNA≥35 Group**	**NBNA<35 Group**	***t***	***P***
	**(*n* = 29)**	**(*n* = 17)**		
IgM(g/L)	0.45 ± 0.15	0.57 ± 0.20	2.409	0.020^*^
IgG(g/L)	7.61 ± 1.41	6.55 ± 1.52	2.378	0.022^*^

* *P < 0.05, which is statistically significant*.

### Craniocerebral MRI and EEG Examination

**Figure 1:**
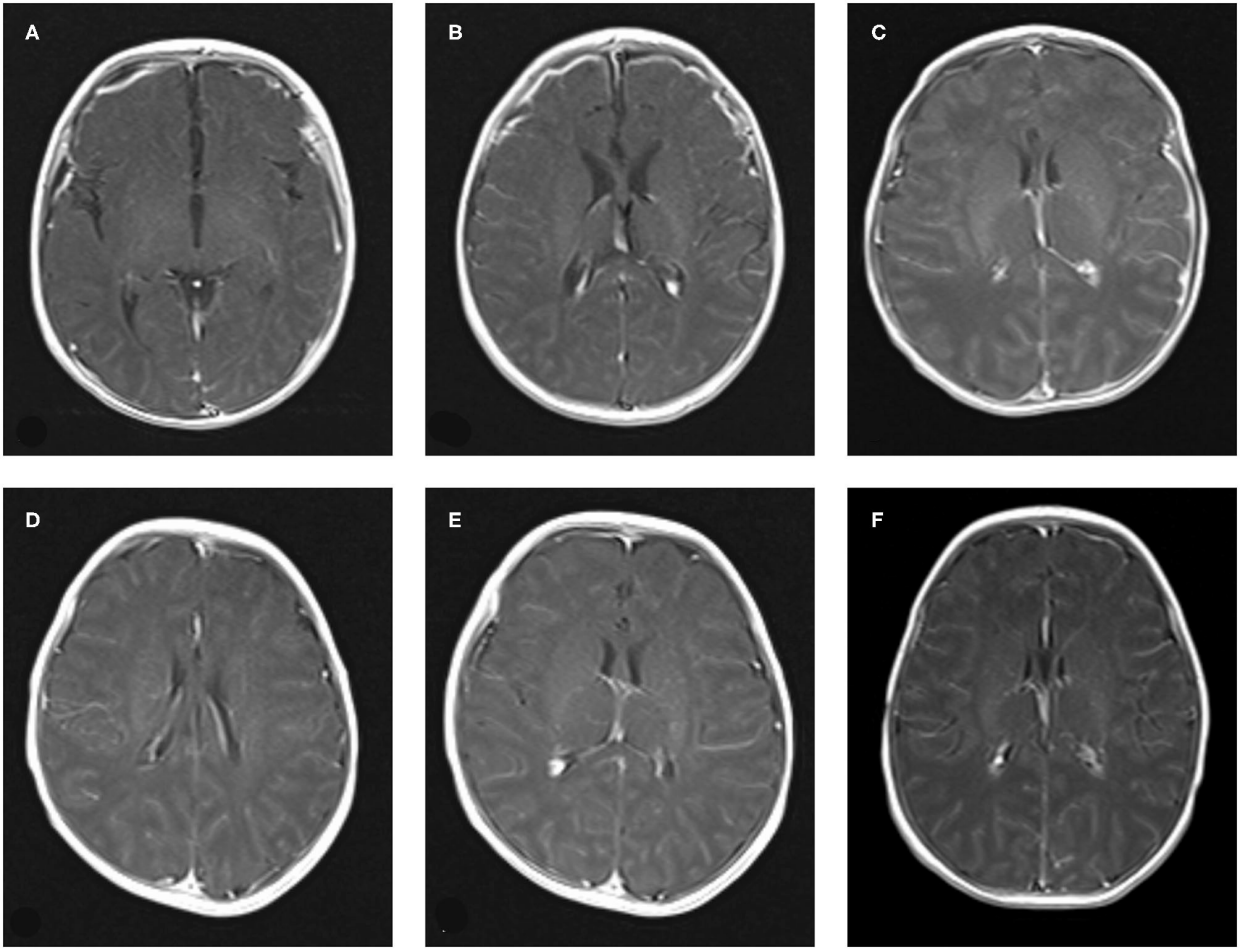
The manifestation of craniocerebral MRI enhanced scan of Neonatal HFMD complicated with encephalitis. **(A–C)**: Bilateral meningeal enhancement. **(D–F)**: The vascular shadow on the surface of the brain increased slightly.

**Figure 2:**
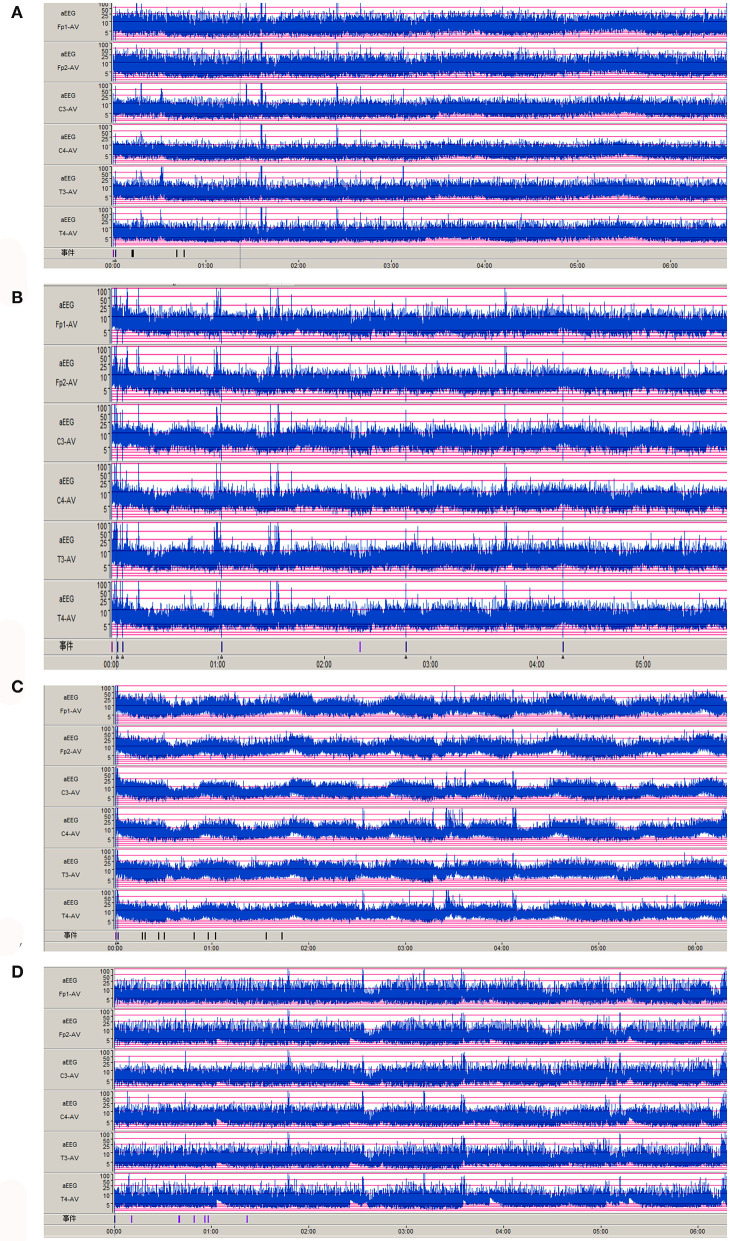
Abnormal aEEG manifestations of Neonatal HFMD complicated with Encephalitis. **(A–D)**: the background activity of aEEG is a discontinuous pattern.

Nine cases of head MRI presented mild abnormalities, mainly manifested as cerebral meningeal enhancement on the brain surface and/or increased cerebral vascular shadows (see Figure 1), including 3 cases in the normal scoring group and 6 cases in the abnormal scoring group. The comparison of abnormal rate of craniocerebral MRI between the normal scoring group and the abnormal scoring group was statistically significant (*P* < 0.05). There were 11 cases of mildly abnormal aEEG, mainly manifested as background activity of EEG: discontinuous graph, of which 5 cases were in the normal group and 6 cases were in the abnormal group. There was no statistically significant difference in the abnormal rate of EEG between the two groups (*P* > 0.05), as shown in the Table 6, Figures 1, 2.

**Table 6:** Comparison of abnormal rate of auxiliary examination for neonates with HFMD and encephalitis.

	**NBNA≥35 Group**	**NBNA<35 Group**	***t***	***P***
	**(*n* = 29)**	**(*n* = 17)**		
Craniocerebral MRI	3	6	4.239	0.040^*^
EEG	5	6	1.604	0.205

* *P < 0.05, which is statistically significant*.

### Prognosis Evaluation

Routine follow-up after discharge showed that 1 case was lost to follow-up in the normal NBNA score group, and all the 28 cases were followed up with a good prognosis. In the abnormal NBNA score group, 1 newborn had a poor prognosis and 16 newborns had a good prognosis.

## Discussion

HFMD is a common infectious disease caused by enterovirus infection. CA16 and enterovirus EV71 are more common, and children are generally susceptible to HFMD. The main source of infection is HFMD and its recessive infection (including adults and children). Neonatal HFMD is rare clinically, but in recent years, neonatal HFMD has an increasing trend year by year (3), mostly in summer. Among the newborns with HFMD complicated with encephalitis in our hospital, most of them were infected with HFMD common virus, a few were infected with CA16 and no EV71 infection, and most of them developed into severe cases because of EV71 infection, which may be one of the reasons for the good prognosis of most of the newborns with HFMD in our hospital. In our hospital, the vast majority of newborns have fever, which is consistent with foreign reports (11). The rash is not specific and atypical, usually manifesting as punctate rash or macular papule. Therefore, for children with summer onset, fever of unknown origin and a clear history of contact, the possibility of HFMD infection should be considered. Foreign studies have reported that the common clinical manifestations of neonatal enterovirus infection are fever, irritation, anorexia, and somnolence (12).

In this study, the clinical characteristics of 46 cases of neonatal HFMD complicated with encephalitis were summarized and analyzed, which were divided into normal NBNA group and abnormal group. There was no significant difference in sex, age, duration of fever, treatment time, rash, respiratory system, digestive system symptoms, signs of intracranial hypertension, muscle tone, and primitive reflex between the two groups (*P* > 0.05). Compared with the state of consciousness, the difference was statistically significant. The main manifestations of abnormal consciousness were drowsiness, irritation, screaming, and no convulsion. aEEG showed that the background activity of EEG was discontinuous and no sharp wave or spike wave was found. The MRI imaging manifestations of meningoencephalitis with different infection degrees are different, so MRI plays an important role in the diagnosis and prognosis of meningitis (13, 14). Contrast-enhanced MRI scan of the neonatal head was mainly manifested as enhanced meningeal signals and increased vascular shadows on the brain surface, without obvious brain parenchymal injury. Therefore, routine follow-up of all newborns after discharge showed that the prognosis was generally good, which was consistent with foreign reports (3).

The level of white blood cell count in CSF can reflect the changes of inflammation exudation in brain tissue. Generally, the higher the white blood cell count, the more severe the inflammatory exudation of the brain tissue and the more serious the central nervous system injury, the more likely it is to develop into a serious disease. In this study, the appearance of CSF in 46 newborn children with HFMD and encephalitis was transparent. The white blood cell count level in the abnormal NBNA score group was higher than that in the normal NBNA score group, and the white blood cell classification was dominated by mononuclear cells. The presence of HFMD associated virus in CSF etiology detection indicated a high positive rate of virus detection in CSF. There was no significant difference in the levels of protein, sugar, chloride and lactate dehydrogenase in CSF between the normal NBNA score group and the abnormal NBNA score group. The change of the biochemical index of CSF in HFMD was not very obvious, which is in accordance to the biochemical characteristics of viral encephalitis.

CK-BB is a brain-type creatine kinase, which usually exists in the heart, skeletal muscle, and brain tissue of the human body. The creatine kinase in the serum of healthy people is mainly skeletal creatine kinase, accounting for about 95% of the total creatine kinase of the human body, brain-type creatine kinase mainly exists in brain tissue cells (neurons and astrocytes), but rarely or even cannot be detected in the serum of healthy people. When brain tissue cells are injured, the content of creatine kinase in CSF is significantly increased. Therefore, it is a sensitive marker of nerve tissue injury, which has high specificity for judging the injury of brain nerve tissue. There is also a close correlation between the increase of CK-BB and the degree of brain injury and prognosis in a variety of nervous system diseases. In this study, the CK-BB level in the CSF of the abnormal NBNA score group was higher than that of the normal NBNA score group. Alkholy et al. (15) studies believe that CK-BB can be used as an important index to evaluate the prognosis of neonatal hypoxic-ischemic encephalopathy, and monitoring the level of CK-BB is helpful to evaluate the condition and prognosis of neonatal hypoxic-ischemic encephalopathy. Therefore, the level of CK-BB in CSF can be used as one of the evaluation indicators for the clinical diagnosis and treatment of neonatal HFMD with encephalitis.

UCH-L1 can be used as a marker for monitoring nerve cell injury in patients with intracranial hemorrhage (16). Previous studies have shown that high levels of UCH-L1 in CSF of patients with craniocerebral trauma indicate a poor prognosis, and the elevated level of serum UCH-L1 is related to the damage of blood-brain barrier and the severity of the disease (17–19). In this study, the level of UCH-L1 in the CSF of the abnormal NBNA score group was higher than that of the normal NBNA score group, suggesting that UCH-L1 may be involved in the pathophysiological process of encephalitis.

Humoral immunity is mediated by B lymphocytes. When stimulated by antigens with the help of TH2 cells, B lymphocytes develop into plasma cells and secrete immunoglobulins. IgM is a pentamer with the largest relative molecular mass. It is the first immunoglobulin synthesized during ontogeny (synthesized in the late embryonic stage). IgM has the largest molecular weight and is the main immunoglobulin produced by the initial humoral immunity, which rises rapidly 4–7 days after birth of normal newborns. In this study, the serum IgM level in the abnormal NBNA group was higher than that in the normal NBNA group, and the increase of IgM level was considered to be related to the early humoral immune response of the body. The content of IgG is the highest in serum and extracellular, and most IgG in neonatal serum comes from the mother, and the amount of self-synthesis is very small. In this study, the serum IgG level in the normal NBNA group was higher than that in the abnormal NBNA group, and the content of neonatal IgG was mainly affected by maternal IgG and gestational age, but was less affected by neonatal itself, suggesting that maternal IgG may play a protective role in the immune response to pathogens. The synthesis and secretion sites of IgA are mainly in the intestinal tract, respiratory tract, mammary gland, salivary gland, and lacrimal gland. IgA participates in mucosal local immunity, and infants can obtain IgA from their mothers' colostrum. In this study, the serum IgA level of newborn children with HFMD was mostly lower than 0.26 g/L, suggesting that the defense ability of gastrointestinal and respiratory mucosa of children with HFMD was decreased.

The overall prognosis of neonatal HFMD complicated with encephalitis is good, which may be due to the protective effect of maternal antibodies in newborns and the immature immune function of newborns without obvious inflammatory storm. There were some differences in clinical manifestations and laboratory indexes compared with preschool children, which did not cause serious clinical manifestations such as brainstem encephalitis, neurogenic pulmonary edema, respiratory failure, heart failure, and so on. However, the sample size of this study is small, and it is still necessary to collect related cases for in-depth analysis.

## Data Availability Statement

The raw data supporting the conclusions of this article will be made available by the authors, without undue reservation, to any qualified researcher.

## Ethics Statement

The studies involving human participants were reviewed and approved by Zhangzhou Hospital affiliated to Fujian Medical University. Written informed consent to participate in this study was provided by the participants' legal guardian/next of kin.

## Author Contributions

YF and CL carried out the studies, participated in collecting data, and drafted the manuscript. DH and LX performed the statistical analysis and participated in its design. All authors read and approved the final manuscript.

## Funding

This study was supported by Startup Fund for scientific research, Fujian Medical University (Grant number: 2018QH1211).

## Conflict of Interest

The authors declare that the research was conducted in the absence of any commercial or financial relationships that could be construed as a potential conflict of interest.
