# Corrigendum: Analysis of clinical related factors of neonatal hand-foot-mouth disease complicated with encephalitis

**DOI:** 10.3389/fneur.2022.945439

**Published:** 2022-09-22

**Authors:** Yanling Fang, Chaowei Lian, Dali Huang, Liping Xu

**Affiliations:** ^1^Zhangzhou Hospital Affiliated to Fujian Medical University, Zhangzhou, China; ^2^Zhangzhou Zhengxing Hospital, Zhangzhou, China

**Keywords:** neonates HFMD, prognosis, risk factors, clinical symptoms, CSF examination, inflammatory factors

In the original article, we neglected to include the funder “Startup Fund for scientific research, Fujian Medical University (Grant number: 2018QH1211).” The correct funding statement appears below.

## Funding

This study was supported by Startup Fund for scientific research, Fujian Medical University (Grant number: 2018QH1211).

The authors apologize for this error and state that this does not change the scientific conclusions of the article in any way. The original article has been updated.

## Publisher's note

All claims expressed in this article are solely those of the authors and do not necessarily represent those of their affiliated organizations, or those of the publisher, the editors and the reviewers. Any product that may be evaluated in this article, or claim that may be made by its manufacturer, is not guaranteed or endorsed by the publisher.

